# Clinically available predictors of obstructive sleep apnoea requiring treatment in type 2 diabetes patients in primary care

**DOI:** 10.1038/s41598-025-93362-1

**Published:** 2025-03-13

**Authors:** Jonas Agholme, Kim Ahtola, Ebba Toll, Carl-Johan Carlhäll, Pontus Henriksson, Stergios Kechagias, Peter Lundberg, Patrik Nasr, Oleg Sysoev, Magnus Wijkman, Mattias Ekstedt, Martin Ulander, Fredrik Iredahl

**Affiliations:** 1 Department of Internal Medicine and Department of Health, Medicine and Caring Sciences, Linköping University, Norrköping, Sweden; 2 Department of Health, Medicine and Caring Sciences, Linköping University, Linköping, Sweden; 3 Department of Clinical Physiology in Linköping, and Department of Health, Medicine and Caring Sciences, Linköping University, Linköping, Sweden; 4 Center for Medical Image Science and Visualization (CMIV), Linköping University, Linköping, Sweden; 5 Department of Gastroenterology and Hepatology and Department of Health, Medicine and Caring Sciences, Linköping University, Linköping, Sweden; 6 Department of Radiation Physics and Department of Health, Medicine and Caring Sciences, Linköping University, Linköping, Sweden; 7 Wallenberg Center for Molecular Medicine, Linköping University, Linköping, Sweden; 8 Division of Statistics and Machine Learning, Department of Computer and Information Science, Linköping University, Linköping, Sweden; 9 Department of Clinical Neurophysiology and Department of Biomedical and Clinical Sciences, Linköping University, Linköping, Sweden; 10 Primary Health Care Center, Department of Health, Medicine and Caring Sciences, Linköping University, Linköping, Sweden

**Keywords:** Pittsburgh sleep quality index, STOP-Bang, Epworth sleepiness scale, Albuminuria, Screening, Diagnostic accuracy, Type 2 diabetes, Epidemiology, Risk factors, Predictive markers, Comorbidities

## Abstract

Obstructive sleep apnoea is a common yet frequently underdiagnosed condition in patients with type 2 diabetes, particularly in primary care. Early detection is important, as untreated sleep apnoea may contribute to worsened metabolic control and increased cardiovascular risk. This study evaluated 164 patients with type 2 diabetes and found that 75% had obstructive sleep apnoea, with 31% requiring treatment for moderate to severe cases. Predicting obstructive sleep apnoea for which medical treatment is indicated (i.e., moderate to severe OSA) proved challenging, as typical clinical symptoms and most other readily available clinical parameters proved to be unreliable indicators. However, central fat distribution, indicated by a higher waist-to-hip ratio (odds ratio 3.31, 95% confidence interval 1.91–6.25, *p* = 0.0032), and the presence of albuminuria (odds ratio 7.46, 95% confidence interval 1.99–27.89, *p* = 0.0244), emerged as significant predictors, with albuminuria representing a novel finding. Screening tools such as the STOP-Bang questionnaire had limited predictive accuracy. These findings highlight the importance of targeted screening in patients with type 2 diabetes, particularly those with central fat distribution or albuminuria, to reduce underdiagnosis and potentially improve treatment outcomes.

## Introduction

Type 2 diabetes mellitus (T2DM) is a prevalent disease, with adult prevalence rates in Nordic countries varying between 3.4–4.4%^1–3^, with a global prevalence near 10%, a trend predicted to increase in the future^4^. Patients with T2DM often have co-morbidities such as dyslipidaemia, overweight/obesity, and/or hypertension^5^, many of which can be easily screened for through readily available blood tests or physical examinations—a crucial part of diabetes management. However, not all co-morbidities are as easily detectable. Obstructive sleep apnoea (OSA), a sleep disorder characterised by snoring and interrupted breathing due to obstruction of the upper airways during sleep^6^, is generally underdiagnosed. In a recent Swedish study of the general population, only a small fraction of those meeting the criteria had a previous OSA diagnosis^7^. This pattern of underdiagnosis is mirrored in patients with T2DM^8^, among whom the prevalence of OSA ranges from 25 to 80%, with milder forms more common than moderate to severe cases^9^. Generally, treatment is indicated for patients with moderate or severe OSA, but not mild^10^. Notably, presence of T2DM increases the risk of incident OSA by up to 50%, irrespective of weight^11^. Conversely, OSA not only increases the risk of developing T2DM but also complicates glucose management independently of weight^12^. The interplay between T2DM and OSA suggests a potential bidirectional pathogenesis and is an area of active research^13^. Authoritative guidelines, such as those from the American Diabetes Association (ADA), acknowledge the importance of evaluating co-existing OSA^14^, and also highlight sleep, independent of OSA, as a key lifestyle factor in T2DM management^15^. Yet, the clinical cues for initiating an OSA evaluation in patients with T2DM remains poorly defined in guidelines and literature.

T2DM and OSA both elevate the risk for secondary conditions, particularly cardiovascular diseases (CVD)^10,15^. When these conditions coexist, the risk of CVD appears to be greater than the sum of their individual risks^16^. To mitigate CVD risk and potentially enhance diabetes management^10^, several leading diabetes organizations advocate screening T2DM patients for OSA^17,18^. However, the high costs and limited accessibility of OSA diagnostic tools, such as polysomnography, pose challenges^10^. While some researchers argue for direct diagnostic evaluation using polysomnography^19^, most screening efforts for patients with T2DM have adopted a two-step approach, beginning with a pre-test probability assessment using more accessible methods, such as questionnaires^20,21^. There is an uncertainty regarding the metabolic and cardiovascular benefits of treating OSA in patients with T2DM, beyond potential effects on resistant hypertension, with discordant results reported in previous studies^10,22^. OSA symptoms, such as daytime sleepiness, can improve with treatment regardless of diabetes status. The uncertainty regarding metabolic and cardiovascular risk reduction is often interpreted as a lack of evidence rather than proof of lacking effect^10^. Consequently, the ethical and economic justification for universal diagnostic evaluation remains uncertain^23^. Meanwhile, guidelines continue to rely on clinical judgement and classical OSA symptoms, such as daytime sleepiness, leaving unresolved the issue of whom to screen.

Consequently, there is a significant knowledge gap regarding how clinicians should approach the assessment of OSA suspicion in an unselected cohort of patients with T2DM. Uncertainty persists concerning the most effective clinical predictive methods for identifying patients at varying risks of OSA, complicating decisions on who should undergo further investigation in this common clinical scenario. This study aims to evaluate and compare the accuracy of various questionnaires and biometric indices when used in the screening of OSA, with the goal of providing clinicians with clearer guidance on assessing OSA risk. Additionally, our research aims to ascertain the prevalence and severity of OSA among patients with T2DM within a primary care setting.

## Methods

### Study design

This study forms part of a population-based cross-sectional observational cohort, the EPSONIP-Sleep sub-study, focusing on the evaluation of OSA in individuals with T2DM. The parent study is the EPSONIP (Evaluating the Prevalence and Severity Of NAFLD in Primary Care) study, which investigates non-alcoholic fatty liver disease (NAFLD) in Swedish primary care settings among people with T2DM. As of 2024, NAFLD is referred to as Metabolic Dysfunction-Associated Steatotic Liver Disease (MASLD). Detailed descriptions of both studies are available in the published study protocol^24^. The study protocol is also registered on clinicaltrials.gov (NCT03864510).

### Setting, recruitment, enrolment and participants

Participants were recruited from primary health care units in Region Östergötland, Sweden, during diabetes follow-up visits between March 2019 and October 2023. Enrolment was continuous, though there was a noticeable decline during the peaks of the COVID-19 pandemic. Eligibility criteria included a diagnosis of T2DM and ages between 35 and 75 years. Exclusions were made for individuals unable to undergo MRI, those with alcohol dependence, diagnosed liver cirrhosis, or primary liver diseases other than MASLD. All participants from the EPSONIP study were invited to join the EPSONIP-Sleep sub-study, except those with implantable cardioverter defibrillators, as their devices could interfere with the Home Sleep Apnoea Test (HSAT) equipment.

### Variables and measurements

#### Outcome

Patients were trained on using the HSAT device (Nox T3, Nox Medical, Reykjavik, Iceland) and equipped themselves with the device for overnight monitoring on the same day. Polygraphic recordings were manually reviewed by a trained physician. Apnoeas were identified as a reduction in nasal flow signal by more than 90% for at least 10 s, while hypopnoeas were defined as a flow reduction of at least 30% for the same duration, accompanied by a minimum desaturation of 3%. AHI was defined as the average number of apnoeas and hypopnoeas per hour of estimated sleep. Sleep was estimated from patient logs and presence of movement artifacts in the recordings. The diagnostic criteria for OSA were categorized as follows: an AHI of < 5/h indicates no OSA; AHI 5/h to < 15/h classify as mild OSA; 15/h to < 30/h as moderate OSA; and ≥ 30/h as severe OSA, adhering to standard practice.

#### Predictors

Predictors were sleep-related questionnaires (Epworth Sleepiness Scale [ESS], Pittsburgh Sleep Quality Index [PSQI], and STOP-Bang). The calculation of total scores from these follows each questionnaire’s specific guidelines, treating total scores as numeric variables based on respondents’ answers. The total score for ESS is the sum of eight items, each assessing the likelihood of dozing off in different situations, indicative of daytime sleepiness^25^. The PSQI, assessing various aspects of sleep quality and disturbances over the last month, involves calculating seven component scores derived from nineteen individual items^26^. These components address subjective sleep quality, sleep latency, duration, habitual efficiency, disturbances, use of sleeping medication, and daytime dysfunction. The total PSQI score, indicating overall sleep quality, is the sum of these components. The STOP-Bang score accumulates points from eight items focusing on snoring, tiredness, observed apnoeas, high blood pressure, body mass index (BMI), age, neck circumference, and sex, and is designed as a screening tool for OSA originally in perioperative patients^27^. Each questionnaire’s total score reflects the severity or presence of sleep-related issues, with higher scores typically indicating poorer sleep quality or higher risk of sleep disorders. Questionnaires were completed by patients prior to the HSAT. The HSAT scorer was blinded regarding the responses to the questionnaires.

Predictor variables also encompassed demographic details such as sex and age, anthropometric data including BMI, waist-to-height ratio, waist-to-hip ratio, clinical measures like blood pressure, HbA1c, haemoglobin, creatinine, diabetes-related complications, lifestyle factors such as physical activity level, smoking, and snus use (a smokeless tobacco product popular in Scandinavia), as well as comorbidities, surgical history, and diabetes-related medication treatment. Research nurses collected all data, recording it directly into case report forms. Blood pressure was measured after 5 min of rest in a seated position. Anthropometric data were collected using a scale and measuring tape. All other predictors were gathered through structured interviews or medical chart reviews.

### Ethics approval and consent to participate

Recruitment processes and the acquisition of written informed consent adhere to national standards, aligning with the Declaration of Helsinki. Data management complies with the General Data Protection Regulation (EU) 2016/679 and meets ICH-GCP guidelines. The study was monitored by Forum Östergötland. The Regional Ethical Board of Östergötland approved the EPSONIP study (2018/176-31 and 2018/494-32) and The Swedish Ethical Review Authority approved the EPSONIP-Sleep extension (2019-03,854).

### Statistical methods

#### Univariate analysis

Student *t*-test and ANOVA were used to assess group differences in numerical means, when data were normally distributed. For non-normally distributed data, Mann–Whitney *u*-test and Kruskal–Wallis H tests were applied. Chi-squared (Χ^2^) or Fisher’s Exact test examined categorical relationships. Predictors for categorical outcomes were analysed using logistic regression, while linear regression was used for numerical outcomes. The Benjamini–Hochberg method corrected for multiple comparisons, with adjusted p-values < 0.05 considered statistically significant.

The strength of the relationship of each global questionnaire score to the outcome variable was computed by thresholding the scores^28^. A binary indicator variable was computed by comparing the scores with a given threshold, and this indicator variable was then compared to the outcome variable to generate true negatives, true positives, false negatives, false positives, sensitivity, and specificity. By varying the threshold value, Receiver Operating Characteristic (ROC) curve with the corresponding Area Under the Curve (AUC) were also computed, along with Positive and Negative Predictive Value (PPV and NPV).

#### Multivariate analysis

Missing values, presumed missing at random, were imputed with multiple imputation by chained equations^29^. The generated datasets were compared to assess variability in the imputed values, and, upon confirming minimal variability, analysis was conducted on a randomly selected imputed dataset. Data were then centered and scaled. Covariates, selected based on clinical relevance and availability, included all variables in Table 1.

**Table 1:** Baseline characteristics by OSA severity in a primary care population of patients with type 2 diabetes mellitus (n = 164).

Variable	Missingness	No OSA	Mild OSA	Moderate OSA	Severe OSA	*p*
	n (%)	41 (25)	72 (43.9)	35 (21.34)	16 (9.75)	
Age, years	0	63.9 (± 7.2)	61.5 (± 9.8)	64.7 (± 5.6)	65.5 (± 8.4)	0.138
Male	0	25 (61.0)	34 (47.2)	26 (74.3)	12 (75.0)	0.026
BMI, kg/m2	5 (3%)	28.8 (± 5.0)	29.5 (± 4.7)	30.4 (± 4.7)	29.7 (± 4.4)	0.516
Waist to height ratio	16 (10%)	0.59 (± 0.06)	0.61 (± 0.07)	0.62 (± 0.07)	0.62 (± 0.07)	0.365
Waist to hip ratio	16 (10%)	0.97 (± 0.07)	0.97 (± 0.07)	1.04 (± 0.09)	1.03 (± 0.07)	< 0.001
Systolic blood pressure, mmHg	3 (2%)	133 (± 14)	131 (± 15)	132 (± 13)	138 (± 12)	0.361
Diastolic blood pressure, mmHg	3 (2%)	80 (± 10)	79 (± 11)	78 (± 12)	80 (± 7)	0.897
Diabetes Duration, years	0	8.9 (± 7.8)	9.1 (± 7.1)	8.0 (± 6.1)	11.5 (± 7.8)	0.438
HbA1c, mmol/mol	0	49.5 (± 7.6)	52.6 (± 10.9)	50.7 (± 11.4)	55.9 (± 12.4)	0.152
HbA1c, %	0	6.7 (± 0.7)	7.0 (± 1.0)	6.8 (± 1.1)	7.3 (± 1.1)	0.152
Haemoglobin, g/l	0	142 (± 11)	141 (± 14)	143 (± 10)	146 (± 15)	0.508
Creatinine, µmol/L	2 (1%)	73 (± 17)	72 (± 18)	74 (± 13)	82 (± 20)	0.200
Smoking	0					0.376
Never smoker		21 (51.2)	34 (47.2)	14 (40.0)	5 (31.2)	
Smoker		3 (7.3)	4 (5.6)	0 (0.0)	2 (12.5)	
Ex-smoker		17 (41.5)	34 (47.2)	21 (60.0)	9 (56.2)	
Snusing	2 (1%)					0.113
Never used snus		25 (61.0)	54 (77.1)	22 (62.9)	11 (68.8)	
Snus user		3 (7.3)	8 (11.4)	7 (20.0)	1 (6.2)	
Ex-snus user		13 (31.7)	8 (11.4)	6 (17.1)	4 (25.0)	
Regular physical activity	4 (2%)	26 (68.4)	34 (47.9)	22 (62.9)	6 (37.5)	0.070
Questionnaire results						
ESS total score	0	6.22 (± 3.42)	6.92 (± 4.02)	5.60 (± 3.81)	5.88 (± 4.36)	0.373
PSQI global score	0	8.29 (± 5.56)	9.32 (± 5.49)	6.14 (± 4.29)	7.31 (± 4.77)	0.029
STOP-bang total score	0	2.98 (± 1.21)	3.50 (± 1.46)	4.20 (± 1.47)	4.50 (± 1.51)	< 0.001
Medical conditions						
Angina	1 (1%)	3 (7.3)	3 (4.2)	3 (8.8)	2 (12.5)	0.603
Myocardial Infarction	1 (1%)	2 (4.9)	5 (6.9)	2 (5.9)	2 (12.5)	0.774
Percutaneous coronary intervention	2 (1%)	2 (4.9)	5 (6.9)	1 (2.9)	2 (13.3)	0.545
Coronary artery bypass graft	1 (1%)	2 (4.9)	1 (1.4)	3 (8.8)	1 (6.2)	0.340
Hypertension	0	24 (58.5)	44 (61.1)	27 (77.1)	12 (75.0)	0.239
Heart Failure	3 (2%)	1/39 (2.5)	2/70 (2.8)	0 (0.0)	1 (6.2)	0.614
Atrial Fibrillation	2 (1%)	1 (2.5)	3 (4.2)	0 (0.0)	1 (6.2)	0.572
Ischemic Stroke	3 (2%)	1 (2.5)	6 (8.5)	1 (2.9)	1 (6.2)	0.514
Hypothyroidism	1 (1%)	4 (10.0)	8 (11.1)	0 (0.0)	2 (12.5)	0.232
Hyperlipidaemia	0	29 (70.7)	50 (69.4)	28 (80.0)	14 (87.5)	0.369
Bariatric surgery	2 (1%)	1 (2.5)	1 (1.4)	0 (0.0)	0 (0.0)	0.762
Diabetic complications						
Current Albuminuria	13 (8%)	3 (8.3)	8 (11.9)	3 (9.1)	7 (46.7)	0.002
Clinical signs of diabetic peripheral neuropathy	12 (8%)	10 (27.8)	11 (16.2)	8 (24.2)	3 (20.0)	0.539
Diabetic retinopathy	12 (8%)	4 (11.1)	13 (19.1)	4 (12.1)	4 (26.7)	0.442
Current medications						
Metformin	1 (1%)	31 (77.5)	58 (80.6)	25 (71.4)	12 (75.0)	0.760
Insulin	6 (4%)	5 (13.2)	17 (24.3)	4 (11.4)	6 (40.0)	0.066
Sulfonylurea	6 (4%)	3 (7.9)	8 (11.4)	0 (0.0)	1 (6.7)	0.225
GLP-1	6 (4%)	1 (2.6)	6 (8.6)	3 (8.6)	0 (0.0)	0.427
SGLT-2	5 (3%)	13 (32.5)	22 (31.4)	8 (23.5)	5 (33.3)	0.816
DPP-4	6 (4%)	0 (0.0)	2 (2.9)	0 (0.0)	0 (0.0)	0.467
Pioglitazone	6 (4%)	0 (0.0)	0 (0.0)	0 (0.0)	0 (0.0)	NaN
Statins	0	31 (75.6)	53 (73.6)	24 (68.6)	13 (81.2)	0.795
Ezetimibe	17 (10%)	1 (2.7)	3 (4.8)	2 (6.1)	2 (14.3)	0.432
Fibrate	18 (11%)	0 (0.0)	0 (0.0)	0 (0.0)	0 (0.0)	NaN

Continuous variables are presented as means (± SD), categorical variables as counts (%), and binary variables by the number of positive cases. Appropriate statistical tests were applied based on the data type: ANOVA for continuous variables, and Chi-squared or Fisher’s exact test for categorical variables.

ANOVA, analysis of variance; BMI, body mass index; DPP-4, dipeptidyl peptidase-4; ESS, epworth sleepiness scale; GLP-1, glucagon-like peptide-1; OSA, obstructive sleep apnoea; PSQI, Pittsburgh sleep quality index; SGLT-2, sodium-glucose transport protein 2.

Stepwise logistic regression, guided by the Akaike Information Criterion, optimized predictor selection for classifying moderate/severe OSA. Iterative refinement excluded non-significant covariates. Leave-One-Out Cross-Validation (LOOCV) was used for statistical validation, with predictions constructing a ROC curve for AUC analysis. Variance Inflation Factor (VIF) analysis addressed multicollinearity among predictors in the final model. Performance metrics, including specificity, sensitivity, PPV, NPV, accuracy, and Cohen’s kappa (κ), were determined using optimal Youden index class probability thresholding.

### Comparisons and stratifications

DeLong’s test evaluated discrimination improvements between the stepwise logistic model and STOP-Bang by comparing ROC AUCs, focusing on rank order differentiation rather than absolute risk. Bootstrap analysis for smaller samples (n < 30) were conducted as a sensitivity analysis, presented only if significant discrepancies emerged in the results.

To account for the limitations of AUC in reflecting clinical utility^30^, we proceeded with Net Reclassification Improvement (NRI) and Integrated Discrimination Improvement (IDI) analyses. NRI analysis enables quantification of the model’s improvement in correctly categorizing patients into risk strata. These thresholds were set at the total prevalence of the outcome ± 20 percentage units, aiming to reflect reasonable probability-based clinical decision making regarding OSA management. The IDI analysis measures the model’s overall improvement in prediction accuracy across all potential risk thresholds. Model performance metrics were computed for clinically relevant thresholds of questionnaire scores. Sensitivity analyses, including sex stratification, assessed the impact of sex differences on predictive capacity. All statistical analyses were conducted in R.

## Results

### Prevalence of OSA and patients characteristics

From the initial 345 participants enrolled in EPSONIP, 18 withdrew consent. Subsequently, 188 consented to join the sleep sub-study, with 183 completing the required questionnaires. Nineteen participants opted out of evaluation with HSAT, resulting in 164 participants ultimately being evaluated in the study.

**Fig. 1:**
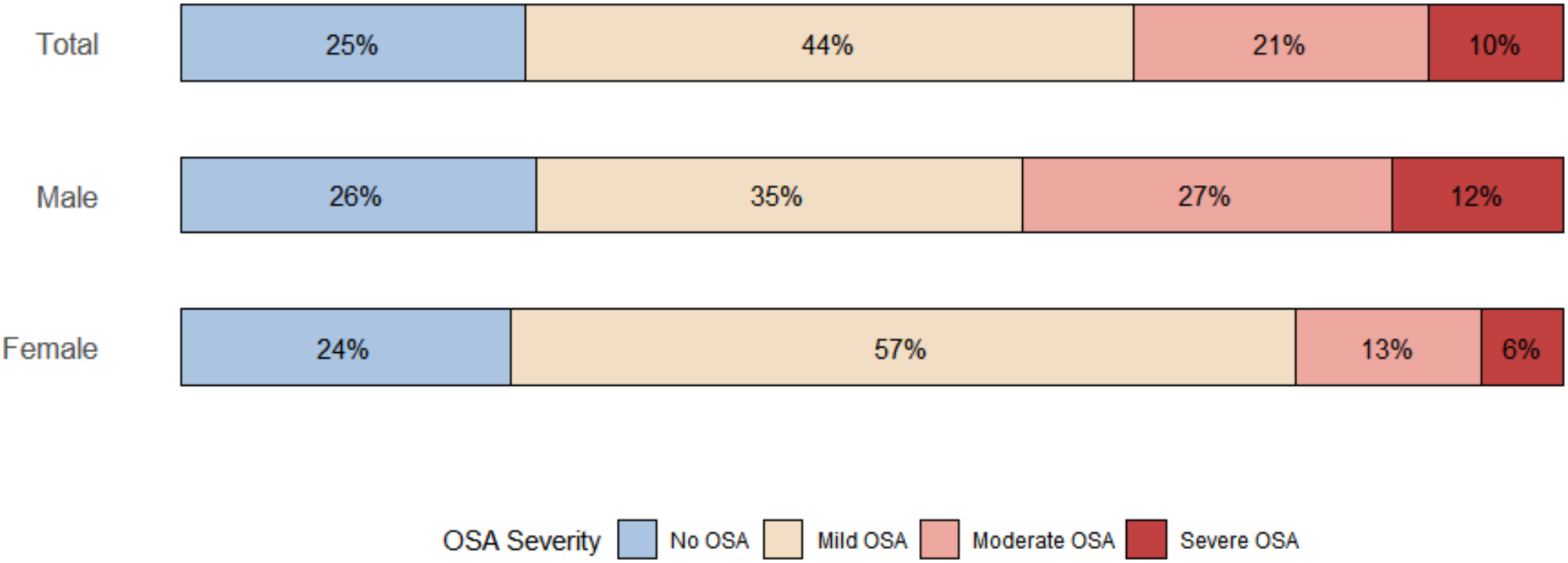
Prevalence of OSA severity among patients with type 2 diabetes mellitus (T2DM) in a primary care setting. The distribution of obstructive sleep apnoea (OSA) severity levels is shown overall and by sex. Severity levels are defined as no OSA (AHI < 5), mild OSA (AHI 5–14), moderate OSA (AHI 15–29), and severe OSA (AHI ≥ 30). The number of individuals in each group are as follows: no OSA—male: 25, female: 16; mild OSA—male: 34, female: 38; moderate OSA—male: 26, female: 9; severe OSA—male: 12, female: 4.

These 164 patients with T2DM were categorized by OSA severity into no OSA (n = 41), mild OSA (n = 72), moderate OSA (n = 35), and severe OSA (n = 16), as shown in Fig. 1. The median age of participants was 65 years, with increasing age correlating with greater OSA severity, and males predominantly occupied the moderate and severe OSA categories, as outlined in Table 1.

Participant characteristics showed no statistically significant differences across study sites, except that participants from Norrköping had slightly higher blood pressure (data not shown). No clinically relevant differences were observed between those who opted to participate and those who did not (data not shown).

### Associations between biometric measures and OSA severity

Biometric univariate analyses, as detailed in Table 2, showed that each 0.1 increase in waist-to-hip ratio was associated with a 5.65 rise in AHI (95% CI 2.88–8.42, BH-adjusted p = 0.0021) and a tripling of the odds for moderate to severe OSA (OR 3.31, 95% CI 1.91–6.25, BH-adjusted p = 0.0032). Albuminuria presented a more pronounced effect, with the presence of albuminuria linked to a more than sevenfold increase in the odds of severe OSA (OR 7.46, 95% CI 1.99–27.89, BH-adjusted p = 0.0244), but the association was not significant for females (BH-adjusted p = 0.285). Given the small number of women with severe OSA (n = 4), we acknowledge that our study was not sufficiently powered to draw definitive conclusions about this subgroup. Specifically, among those with severe OSA, 7 had albuminuria and 8 did not, while in the other categories, 14 had albuminuria and 122 did not.

**Table 2:** Univariate associations with polysomnographic measures.

Predictor	Outcome	Method	Estimate (95% CI)	*P*-value adjusted
Waist to hip ratio (0.1)	Moderate or severe OSA	Logistic regression	OR 3.31 (1.91–6.25)	0.0032
Total score of STOP-bang	Moderate or severe OSA	Logistic regression	OR 1.61 (1.23–2.09)	0.004
Total score of ESS	Moderate or severe OSA	Logistic regression	OR 0.93 (0.85–1.02)	0.4202
Total score of PSQI	Moderate or severe OSA	Logistic regression	OR 0.91 (0.84–0.97)	0.0609
Total score of STOP-bang	Actual AHI	Linear regression	β 3.16 (2.88–8.41)	0.0004
Waist to hip ratio (0.1)	Actual AHI	Linear regression	β 5.64 (2.88–8.42)	0.0021
Albuminuria	Severe OSA	Logistic regression	OR 7.46 (1.99–27.89)	0.0244

Associations that have undergone Benjamini–Hochberg adjustment, along with their respective estimates pre-adjustment. Waist-to-hip ratio values have been scaled down by a factor of 10 to depict more realistic variations.

AHI, apnoea-hypopnoea index; ESS, epworth sleepiness scale; PSQI, Pittsburgh sleep quality index; OSA, obstructive sleep apnoea.

**Fig. 2:**
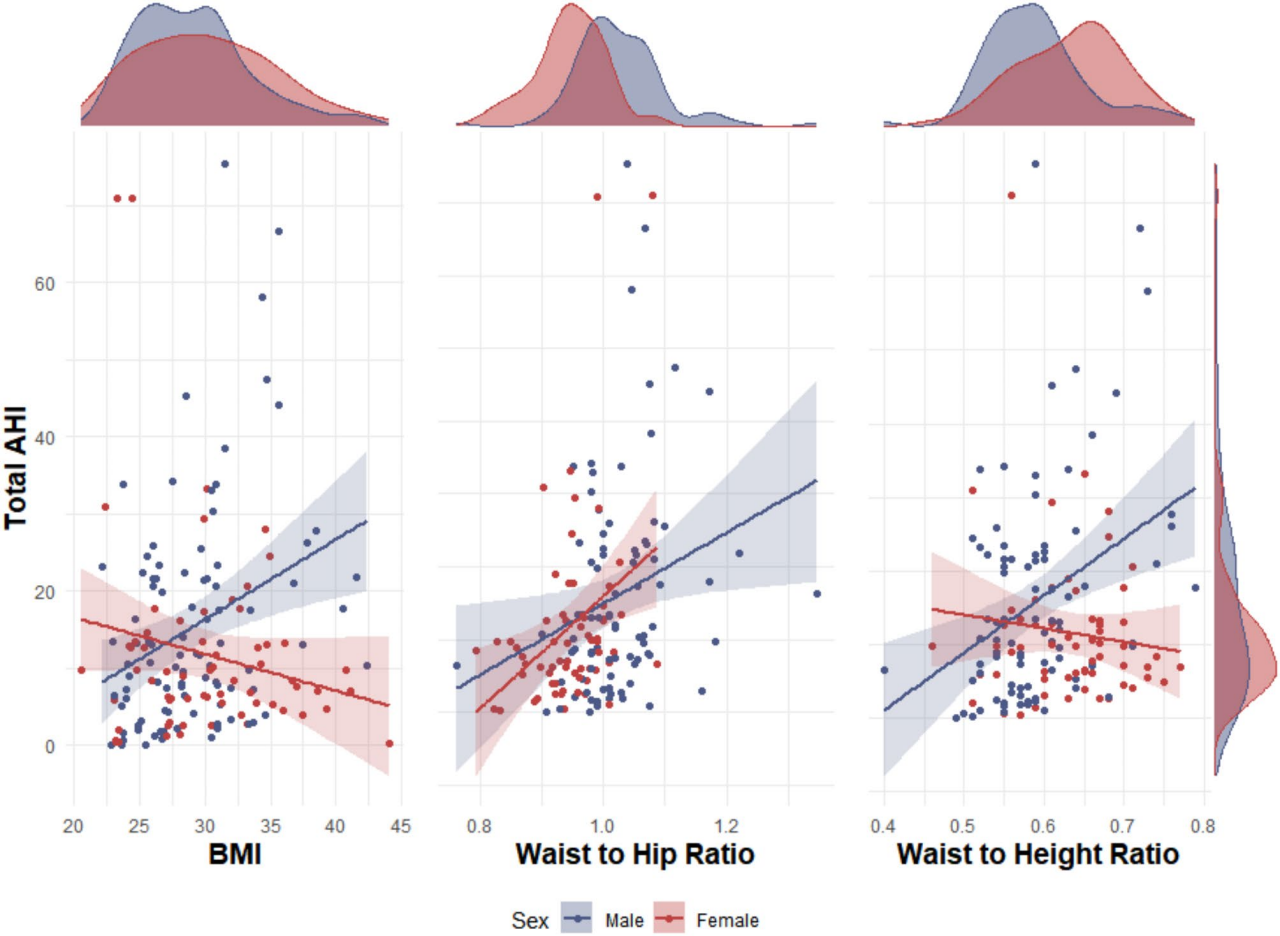
Relationship between the total Apnoea-Hypopnea Index (AHI) and anthropometric metrics—BMI, waist-to-hip ratio, and waist-to-height ratio—across sexes. Each plot is detailed with linear model fit lines, confidence intervals, and accompanying density plots that illustrate the distribution of each metric. BMI shows a significant positive correlation with total AHI in males (β = 1.03, p = 0.002) but not in females (β = -0.47, p = 0.129). The waist-to-hip ratio indicates a positive significant relationship with total AHI in both males (β = 48.83, p = 0.023) and females (β = 77.00, p = 0.006). The waist-to-height ratio demonstrates a significant positive correlation in males (β = 77.60, p = 0.001) but not in females (β = -18.36, p = 0.390).

Sex-stratified analyses revealed differing relationships between anthropometric measures and OSA severity. For males, higher BMI, waist-to-hip ratio, and waist-to-height ratio were associated with increased AHI scores (Fig. 2). In contrast, females showed no significant correlation between BMI, waist-to-height ratio, and AHI scores, while waist-to-hip ratio’s association with AHI and moderate or severe OSA was similar to that in males (Fig. 2). Interaction terms between sex and these anthropometric measures were tested. The interaction between sex and BMI was statistically significant (p = 0.0011), as was the interaction between sex and waist-to-height ratio (p = 0.0035). However, the interaction between sex and waist-to-hip ratio was not statistically significant (p = 0.4299).

### Predictive values of questionnaires

**Fig. 3:**
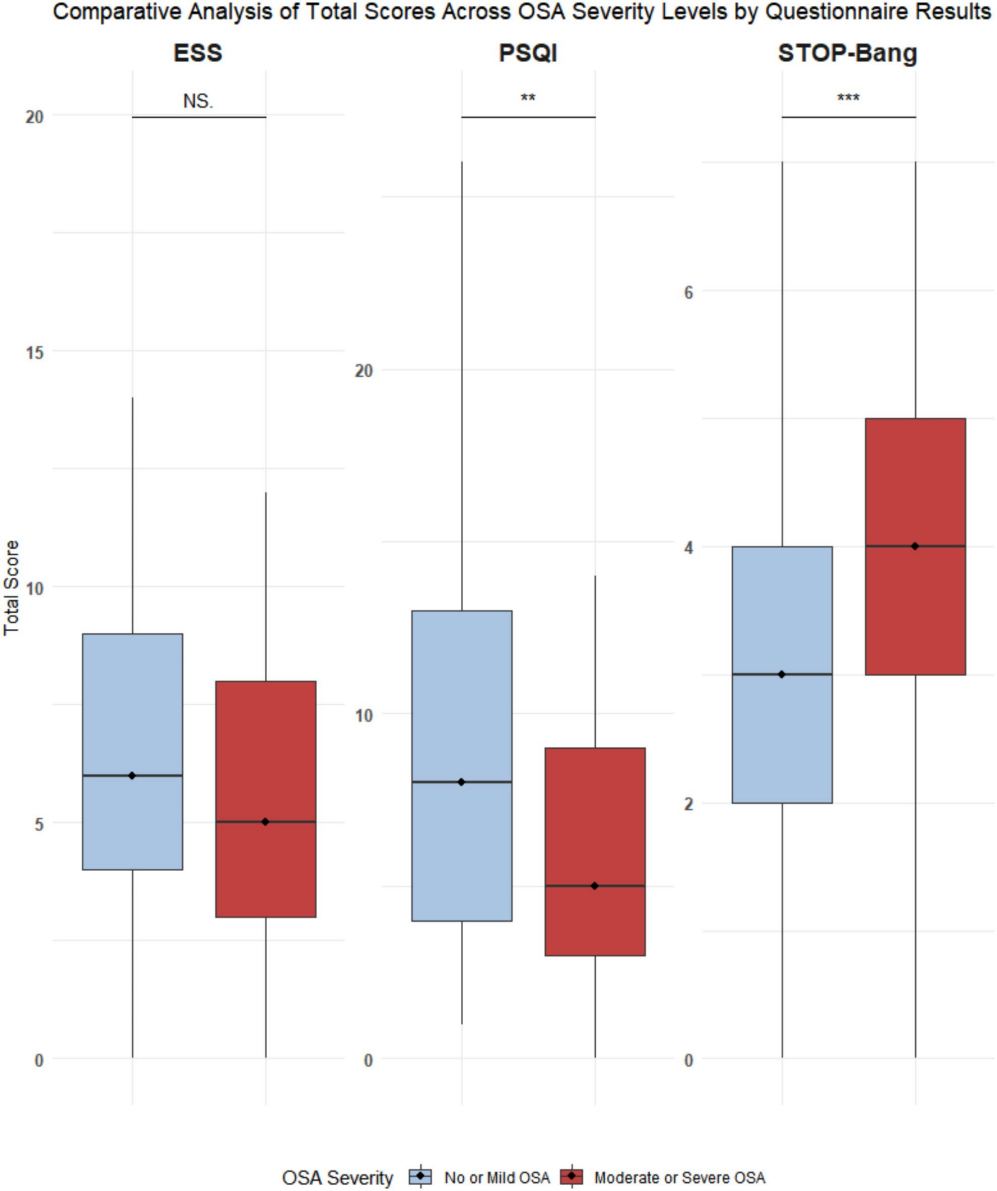
Comparative analysis of total scores across OSA severity levels by questionnaire results. The distribution of total scores from three questionnaires—Epworth Sleepiness Scale (ESS, max score 24), Pittsburgh Sleep Quality Index (PSQI, max score 21), and STOP-Bang (max score 8)—categorized by obstructive sleep apnoea (OSA) severity. Each boxplot shows the median (horizontal line), interquartile range (IQR, box), and data spread (whiskers extending to 1.5 * IQR from the hinges). Statistically significant differences between ‘No or Mild OSA’ and ‘Moderate or Severe OSA’ categories were assessed using Mann–Whitney U tests. Significance levels are indicated above comparisons. * indicates a p-value less than 0.05, ** indicates a p-value less than 0.01, and *** indicates a p-value less than 0.001. Number of measurements: No OSA: n = 113, Moderate or Severe OSA: n = 51 for all questionnaires.

Figure 3 presents the distribution of the questionnaire scores. As shown in Table 2, STOP-Bang demonstrated a significant post-adjustment association with moderate to severe OSA, indicated by an OR of 1.61 (95% CI 1.24–1.74, BH-adjusted p = 0.004), implying 61% higher odds for each score increment. Correspondingly, AHI increased by 3.16 units per STOP-Bang point (95% CI 1.80–4.52, p = 0.0004). PSQI, inversely associated with OSA odds, decreased by 9% per point (OR 0.91, 95% CI 0.84–0.97), but lost significance after BH-adjustment. ESS showed an inverse relationship without significance (p = 0.136).

### Performance metrics of stepwise logistic regression

**Fig. 4:**
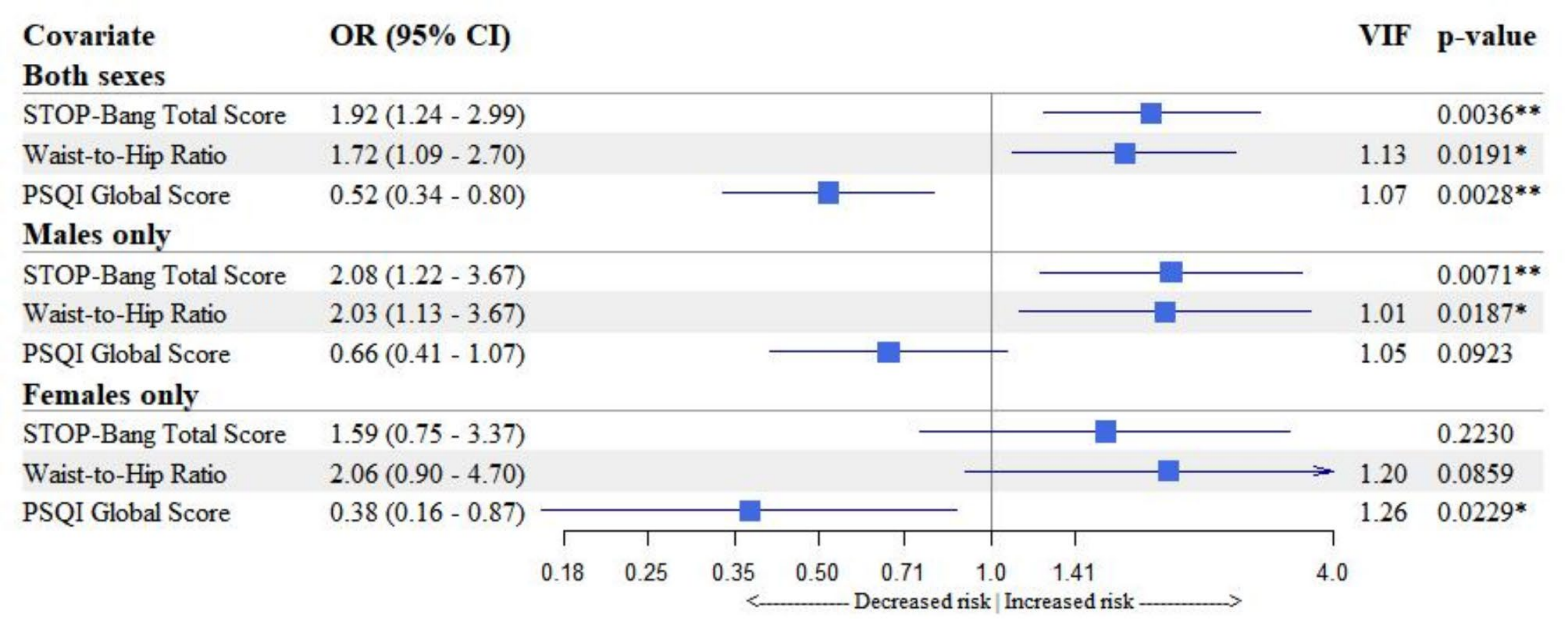
Forest plot of the best predictive model for moderate or severe obstructive sleep apnoea. Associations between predictors and moderate or severe OSA were derived from stepwise logistic regression, both overall and by sex. Covariates, including total scores of STOP-Bang and PSQI, as well as the waist-to-hip ratio, were standardized. Log-transformed odds ratios and their 95% confidence intervals for each variable are presented both graphically and numerically. These odds ratios estimate the risk associated with a one-unit change in the standardized variable. Variance Inflation Factor values were calculated to assess potential multicollinearity among covariates, with values exceeding 5 indicating multicollinearity. Abbreviations: OR, Odds Ratio; CI, Confidence Interval; VIF, Variance Inflation Factor; PSQI, Pittsburgh Sleep Quality Index; OSA, Obstructive Sleep Apnoea.

The stepwise logistic model evaluated all variables from Table 1, with the best predictive model presented in Fig. 4. It identified the STOP-Bang total score (OR 1.92, 95% CI 1.24–2.99, p = 0.004), PSQI total score (OR 0.52, 95% CI 0.34–0.80, p = 0.003), and waist-to-hip ratio (OR 1.72, 95% CI 1.09–2.70, p = 0.019) as significant predictors of moderate or severe OSA. The analysis also included a sensitivity check across sexes and all potential interaction terms with previously described confounders like age, sex, BMI, and hypertension. None of the interaction terms resulted in any significant changes to the model.

The model achieved 72% accuracy in differentiating moderate to severe OSA, with sensitivity at 62.7%, specificity at 76.1%, PPV at 54.2%, and NPV at 81.9%, against a 31.1% prevalence and 19.5% detection rate, using a threshold value of 0.5. Balanced accuracy was 69.4%, with a kappa statistic of 0.372 and an AUC of 0.70. Compared to STOP-Bang alone, DeLong’s test was non-significant, the NRI demonstrated a 32.88% enhancement in correct reclassification (95% CI 15.91–49.85%, p = 0.0001), and the IDI showed an 10.60% increase (95% CI 5.36–15.83%, p = 0.0001) in distinguishing between patients’ predicted probabilities.

## Discussion

Our study identified waist-to-hip ratio and albuminuria as key clinical predictors of OSA in patients with T2DM. These factors stood out as reliable biometric indicators of moderate to severe OSA, suggesting that patients who exhibit either a high waist-to-hip ratio or the presence of albuminuria should be considered for OSA evaluation, regardless of the presence of typical symptoms. This finding aligns with the broader understanding that waist-to-hip ratio is a strong biometric predictor of OSA severity across both sexes, while other measures such as BMI and waist-to-height ratio exhibit varying distribution profiles between sexes^31^. In the general female population, menopause increases OSA risk^32^, correlating with a shift towards male-pattern central fat distribution^33^. In our cohort, over 90% of women were likely post-menopausal, being older than 55, yet disparities in the predictive value of anthropometric measures persisted, implying that measurements including waist circumference, such as waist-to-hip ratio, are pragmatic for accurately assessing OSA risk in women with T2DM.

The association between severe OSA and albuminuria, possibly indicative of early diabetic kidney disease, aligns with findings from Spain showing over 50% prevalence of severe OSA among patients with diabetic kidney disease^34^. There is evidence suggesting a bidirectional pathogenesis where intermittent hypoxia during sleep exacerbates kidney function deterioration, and fluid retention, in turn, aggravates OSA^35,36^. This highlights a complex, clinically significant interplay between T2DM, OSA, and kidney disease, where each condition may aggravate the others. Targeted interventions to disrupt this cycle could be of considerable clinical value. Given the suggested substantial overlap and potentially severe consequences of OSA in this demographic, maintaining a low threshold for screening patients with diabetic kidney disease is advisable. Our study’s power likely was insufficient to assess the relevance in females, due to the low prevalence of both severe OSA and albuminuria in this group. Thus, while our findings suggest a strong association between albuminuria and severe OSA overall, the relevance of this relationship in women remains uncertain and requires further investigation.

The STOP-Bang questionnaire’s direct correlation with an increased likelihood of moderate to severe OSA, evidenced by an AUC of 0.69, corroborates its utility as a practical screening tool in primary care. This performance was lower than that observed in Chinese patients with T2DM (AUC 0.86)^37^, yet slightly superior when comparing sensitivity, specificity, and other metrics to a Czech study targeting a similar population^19^. Outside the T2DM context, the STOP-Bang questionnaire exhibits a performance characterised by high sensitivity, efficiently identifying individuals with OSA but consequently higher false positives^38^.

Our analysis found that classical OSA symptoms, including daytime sleepiness (ESS) and poor sleep quality (PSQI), had limited value in predicting OSA in patients with T2DM. The inverse association observed with between the PSQI scores and OSA severity challenges the assumption that poorer subjective sleep quality is indicative of more severe disease. One possible explanation is how the PSQI operationalizes sleep quality. Higher scores indicate problems such as difficulty falling asleep and short sleep duration. However, these issues are less common in severe OSA, where patients often fall asleep quickly and sleep longer due to increased sleep pressure from repeated nighttime arousals.

In contrast, within the general population in a primary care context, the ESS shows better performance in identifying OSA^39,40^ despite its low specificity; OSA patients tend to report higher ESS scores^41^. T2DM patients show elevated fatigue levels irrespective of OSA status^42^, complicating symptom attribution to OSA without evidence of post-treatment improvement. This situation blurs the line between symptomatic and asymptomatic OSA in T2DM, a crucial distinction given that OSA patients with sleepiness or fatigue face a potentially doubled risk of CVD compared to those with similar symptoms but no OSA diagnosis^43^.

Stepwise regression analysis showed that adding PSQI and waist-to-hip ratio to the STOP-Bang questionnaire improved the accuracy of classifying obstructive sleep apnoea risk, as evidenced by significant NRI and IDI values. However, this combination did not significantly enhance the overall predictive accuracy of the model’s AUC compared to STOP-Bang alone. This suggest that while overall model performance as measured by AUC remained similar, the addition of new predictors improved the model’s ability to accurately categorize patients into more precise risk strata^30^.

In practical terms, this means that for primary care clinicians, simply relying on STOP-Bang alone may provide a broad but imprecise risk estimate, while incorporating waist-to-hip ratio and PSQI may allow for a more nuanced risk assessment. Similar studies using complex variables, such as the homeostasis model assessment insulin resistance index and MASLD evaluation, have also shown only marginal improvements in AUC^44^, indicating that simpler, accessible measures might be just as effective in primary care settings. However, it also highlights the current lack of clinical markers that can truly enhance the discriminatory ability to detect obstructive sleep apnoea in patients with type 2 diabetes.

The potential cardiovascular and metabolic benefits of OSA treatment in patients with T2DM remain an area of ongoing debate. Large RCTs, such as the SAVE trial, have not demonstrated a significant reduction in major cardiovascular events with CPAP treatment in unselected populations with established cardiovascular disease^45^. However, recent meta-analyses suggest that CPAP may provide benefits in high-risk subgroups, particularly in patients with coronary artery disease and good adherence to therapy^46^. Specifically, CPAP use exceeding 4 h per night was associated with a 23% reduction in all-cause and cardiovascular mortality in these patients. The relevance of these findings to patients with T2DM remains uncertain, and further research is needed to determine whether OSA can be considered a modifiable risk factor for diabetes-related outcomes, such as CVD, as highlighted by the recently published International Consensus Statement on OSA^10^.

Given that treatment adherence is a key determinant of risk reduction, it is particularly relevant to consider adherence in patients with T2DM, as many with moderate to severe OSA in our study reported few or no classical OSA symptoms. Prior research suggests that screened patients with T2DM can achieve promising initial adherence levels, with 39% still using CPAP after one year^47^. While this rate leaves room for improvement, it indicates that with appropriate education and support, even patients who do not initially perceive symptoms may still accept and adhere to CPAP treatment.

Our study revealed a strikingly high prevalence of obstructive sleep apnoea in Swedish primary care patients with type 2 diabetes, affecting approximately 75% of the cohort, with one-third potentially needing treatment. This significant overlap between the two conditions underscores the urgent need for more effective screening strategies that go beyond clinical judgment or subjective symptom assessment, instead leveraging objective measures to better identify at-risk patients. Notably, despite commonly reported sex disparities, our findings suggest a uniform prevalence of obstructive sleep apnoea across sexes in this population, although men were more likely to experience severe forms. This is in concordance with existing literature, indicating that men are more susceptible to OSA compared to women in the target population^19,48^. The variability in reported prevalence of moderate to severe obstructive sleep apnoea among patients with type 2 diabetes in previous studies—ranging from 23.8 to 53%—likely reflects differences in cohort characteristics, such as BMI, as well as evolving diagnostic criteria and demographic factors like ethnicity and geographical location^48–51^.

Future large-scale prospective studies or randomized trials are needed to determine whether OSA is a truly modifiable risk factor for major diabetic complications, including cardiovascular disease, nephropathy, and poor metabolic control. If evidence emerges that OSA treatment provides significant benefits primarily to specific high-risk subgroups such as those with central obesity, albuminuria, or advanced cardiovascular risk, this would have direct implications for screening strategies. Rather than advocating universal OSA evaluation in all T2DM patients, a more targeted approach could be adopted, prioritizing high-risk individuals for diagnostic testing and intervention. Such a strategy could optimize resource allocation and improve clinical outcomes by focusing on those most likely to benefit from treatment.

### Strengths and limitations

This study’s strengths include its representation of primary care patients with T2DM with modern treatment, characterised by a broad spectrum of patients with generally low comorbidity and complication frequencies, enhancing our findings’ external validity.

Despite these strengths, certain limitations warrant consideration. The cross-sectional nature of the study limits our ability to deduce causal relationships or the directionality of the observed associations but does not preclude the validity of the associations as a predictor. The lack of detailed ethnicity data, due to legal restrictions, prevents examination of how ethnic diversity within Sweden might influence OSA prevalence and characteristics in the T2DM population. Although no signs of selection bias were evident among participants progressing to the sub-study, the absence of systematic documentation for non-participation introduces potential for bias, especially from patients with pre-diagnosed OSA who might have opted out. Potential biases might arise from the non-participation of such individuals with pre-diagnosed OSA and the inherited exclusion of those with alcohol usage or known liver disease, known risk factors for OSA and thus underestimating the true prevalence of OSA. Yet, these exclusions are likely to have minimal effect on generalizability and, if anything, might suggest conservative prevalence estimates. Despite potential for selection bias and the study’s limitations in capturing all nuances, these factors are unlikely to affect the primary or secondary aims significantly.

Moreover, the original EPSONIP study was designed with sufficient power to determine the prevalence of advanced liver fibrosis, a condition less common than OSA. Consequently, a specific power calculation for the sleep sub-study was not performed. Increasing the sample size might have improved the identification of variations within subgroups in primary care, potentially uncovering nuanced associations, such as the role of albuminuria in females, which may have been missed due to type II errors.

Furthermore, our study used HSAT rather than polysomnography, which is the golden standard. In the Nordic countries, a pathologic HSAT is typically sufficient to initiate OSA treatment in patients where it is deemed necessary^52^, and polysomnography is only performed in unclear cases, which is why the study was based on HSAT. This may not be true for all countries, however, and it might affect the generalizability of the findings.

## Conclusion

Detection of OSA in patients with T2DM is complicated by the low predictive value of classical symptoms. However, the presence of albuminuria or a high waist-to-hip ratio strongly indicates a higher likelihood of needing treatment for OSA. While STOP-Bang has some utility in screening, its predictive capacity is limited, and neither ESS nor PSQI showed accuracy in this population. The significant overlap between OSA and T2DM, highlighted by the high prevalence observed and the widespread underdiagnosis of OSA, underscores an urgent need to determine whether OSA treatment can positively impact clinical outcomes in T2DM. If so, future efforts should focus on developing more robust biomarkers or considering universal diagnostic evaluation, given the current limitations of pre-screening tools.

## Data Availability

The datasets generated and analysed during the current study are not publicly available due to the sensitive nature of personal data and the requirement for ethical approval from The Swedish Ethical Review Authority for the data to be legally shared, but are available from the corresponding author on reasonable request and with a valid ethical permit.

## Abbreviations

ADA: American Diabetes Association
AHI: Apnoea–Hypopnoea Index
AUC: Area under the curve
BMI: Body mass index
CVD: Cardiovascular disease
ESS: Epworth sleepiness scale
HSAT: Home sleep apnoea test
ICH-GCP: International conference on harmonisation of good clinical practice
IDI: Integrated discrimination improvement
LOOCV: Leave-one-out cross-validation
MASLD: Metabolic dysfunction associated steatotic liver disease
MRI: Magnetic resonance imaging
NAFLD: Non-alcoholic fatty liver disease
NRI: Net reclassification improvement
NPV: Negative predictive value
OSA: Obstructive sleep apnoea
PPV: Positive predictive value
PSQI: Pittsburgh sleep quality index
ROC: Receiver operating characteristic
T2DM: Type 2 diabetes mellitus
VIF: Variance inflation factor

## References

[1] Carstensen, B. & Rønn, P. F. Jørgensen ME (2020) Prevalence, incidence and mortality of type 1 and type 2 diabetes in Denmark 1996–2016. *BMJ Open Diabetes Res. Care* **8**, e001071 (2020). 32475839 10.1136/bmjdrc-2019-001071 PMC7265004

[2] Slåtsve, K. B. et al. The total prevalence of diagnosed diabetes and the quality of diabetes care for the adult population in Salten, Norway. *Scand. J. Public Health* **50**, 161–171 (2022). 32854596 10.1177/1403494820951004 PMC8873303

[3] Veyhe, A. S. et al. Prevalence of prediabetes and type 2 diabetes in two non-random populations aged 44–77 years in the Faroe Islands. *J. Clin. Transl. Endocrinol.* **16**, 100187 (2019). 31032180 10.1016/j.jcte.2019.100187 PMC6477859

[4] Sun, H. et al. IDF Diabetes Atlas: Global, regional and country-level diabetes prevalence estimates for 2021 and projections for 2045. *Diabetes Res. Clin. Pract.* **183**, 109119 (2022). 34879977 10.1016/j.diabres.2021.109119 PMC11057359

[5] Iglay, K. et al. Prevalence and co-prevalence of comorbidities among patients with type 2 diabetes mellitus. *Curr. Med. Res. Opin.* **32**, 1243–1252 (2016). 26986190 10.1185/03007995.2016.1168291

[6] Sateia, M. J. International classification of sleep disorders-third edition: Highlights and modifications. *Chest* **146**, 1387–1394 (2014). 25367475 10.1378/chest.14-0970

[7] Baldanzi, G. et al. OSA is associated with the human gut microbiota composition and functional potential in the population-based Swedish cardiopulmonary bioimage study. *CHEST* **164**, 503–516 (2023). 36925044 10.1016/j.chest.2023.03.010 PMC10410248

[8] Heffner, J. E., Rozenfeld, Y., Kai, M., Stephens, E. A. & Brown, L. K. Prevalence of diagnosed sleep apnea among patients with type 2 diabetes in primary care. *Chest* **141**, 1414–1421 (2012). 22095313 10.1378/chest.11-1945

[9] Ogilvie, R. P. & Patel, S. R. The epidemiology of sleep and diabetes. *Curr. Diab. Rep.* **18**, 82 (2018). 30120578 10.1007/s11892-018-1055-8 PMC6437687

[10] Chang, J. L. et al. International consensus statement on obstructive sleep apnea. *Int. Forum Allergy Rhinol.* **13**, 1061–1482 (2023). 36068685 10.1002/alr.23079 PMC10359192

[11] Subramanian, A. et al. Risk of incident obstructive sleep apnea among patients With Type 2 diabetes. *Diabetes Care* **42**, 954–963 (2019). 30862657 10.2337/dc18-2004

[12] Kent, B. D. et al. Diabetes mellitus prevalence and control in sleep-disordered breathing: The European Sleep Apnea Cohort (ESADA) study. *Chest* **146**, 982–990 (2014). 24831859 10.1378/chest.13-2403

[13] Framnes, S. N. & Arble, D. M. The bidirectional relationship between obstructive sleep apnea and metabolic disease. *Front. Endocrinol.* **9**, 440 (2018). 10.3389/fendo.2018.00440 PMC6087747 30127766

[14] American Diabetes Association. 3. Initial evaluation and diabetes management planning. *Diabetes Care* **38**, S17–S19 (2015). 25537701 10.2337/dc15-S006

[15] Davies, M. J. et al. Management of hyperglycaemia in type 2 diabetes, 2022. A consensus report by the American Diabetes Association (ADA) and the European Association for the Study of Diabetes (EASD). *Diabetologia* **65**, 1925–1966 (2022). 36151309 10.1007/s00125-022-05787-2 PMC9510507

[16] Paschou, S. A. et al. Sleep apnea and cardiovascular risk in patients with prediabetes and type 2 diabetes. *Nutrients* **14**, 4989 (2022). 36501019 10.3390/nu14234989 PMC9741445

[17] International Diabetes Federation. The IDF consensus statement on sleep apnoea and type 2 diabetes. (2008).

[18] Viswanathan, V., Ramakrishnan, N., Saboo, B. & Agarwal, S. RSSDI clinical practice recommendations for screening, diagnosis, and treatment in type 2 diabetes mellitus with obstructive sleep apnea. *Int. J. Diabetes Dev. Ctries.* **41**, 4–21 (2021).

[19] Westlake, K., Plihalova, A., Pretl, M., Lattova, Z. & Polak, J. Screening for obstructive sleep apnea syndrome in patients with type 2 diabetes mellitus: A prospective study on sensitivity of Berlin and STOP-Bang questionnaires. *Sleep Med.* **26**, 71–76 (2016). 27613528 10.1016/j.sleep.2016.07.009

[20] Donovan, L. M. et al. The effectiveness of an obstructive sleep apnea screening and treatment program in patients with type 2 diabetes. *Diabetes Res. Clin. Pract.* **134**, 145–152 (2017). 29054482 10.1016/j.diabres.2017.10.013 PMC5724386

[21] Morrison, H. B., Padilla, B. I., Thompson, J. A. & Kreider, K. E. Obstructive sleep apnea and type 2 diabetes: A screening approach. *J. Nurse Pract.* **18**, 580–582 (2022).

[22] Abud, R. et al. Efficacy of continuous positive airway pressure (CPAP) preventing type 2 diabetes mellitus in patients with obstructive sleep apnea hypopnea syndrome (OSAHS) and insulin resistance: A systematic review and meta-analysis. *Sleep Med.* **62**, 14–21 (2019). 31518943 10.1016/j.sleep.2018.12.017

[23] Jonas, D. E. et al. Screening for obstructive sleep apnea in adults: Evidence report and systematic review for the US preventive services task force. *JAMA* **317**, 415 (2017). 28118460 10.1001/jama.2016.19635

[24] Nasr, P. et al. Evaluating the prevalence and severity of NAFLD in primary care: The EPSONIP study protocol. *BMC Gastroenterol.* **21**, 180 (2021). 33879084 10.1186/s12876-021-01763-z PMC8056630

[25] Johns, M. W. A new method for measuring daytime sleepiness: The epworth sleepiness scale. *Sleep* **14**, 540–545 (1991). 1798888 10.1093/sleep/14.6.540

[26] Buysse, D. J., Reynolds, C. F., Monk, T. H., Berman, S. R. & Kupfer, D. J. The Pittsburgh sleep quality index: A new instrument for psychiatric practice and research. *Psychiatry Res.* **28**, 193–213 (1989). 2748771 10.1016/0165-1781(89)90047-4

[27] Chung, F. et al. STOP questionnaire: A tool to screen patients for obstructive sleep apnea. *Anesthesiology* **108**, 812–821 (2008). 18431116 10.1097/ALN.0b013e31816d83e4

[28] Hanley, J. A. & McNeil, B. J. The meaning and use of the area under a receiver operating characteristic (ROC) curve. *Radiology* **143**, 29–36 (1982). 7063747 10.1148/radiology.143.1.7063747

[29] Buuren, S. V. & Groothuis-Oudshoorn, K. Mice : Multivariate imputation by chained equations in R. *J. Stat. Softw.* **45,** (2011).

[30] Cook, N. R. Quantifying the added value of new biomarkers: How and how not. *Diagn. Progn. Res.* **2**, 14 (2018). 31093563 10.1186/s41512-018-0037-2 PMC6460632

[31] Mazzuca, E. et al. Gender-specific anthropometric markers of adiposity, metabolic syndrome and visceral adiposity index (VAI) in patients with obstructive sleep apnea. *J. Sleep Res.* **23**, 13–21 (2014). 24118617 10.1111/jsr.12088

[32] Young, T., Finn, L., Austin, D. & Peterson, A. Menopausal status and sleep-disordered breathing in the Wisconsin sleep cohort study. *Am. J. Respir. Crit. Care Med.* **167**, 1181–1185 (2003). 12615621 10.1164/rccm.200209-1055OC

[33] Ambikairajah, A., Walsh, E., Tabatabaei-Jafari, H. & Cherbuin, N. Fat mass changes during menopause: A metaanalysis. *Am. J. Obstet. Gynecol.* **221**, 393-409.e50 (2019). 31034807 10.1016/j.ajog.2019.04.023

[34] Zamarrón, E. et al. Obstructive sleep apnea is associated with impaired renal function in patients with diabetic kidney disease. *Sci. Rep.* **11**, 5675 (2021). 33707611 10.1038/s41598-021-85023-w PMC7952421

[35] Lin, C.-H., Lurie, R. C. & Lyons, O. D. Sleep apnea and chronic kidney disease: A state-of-the-art review. *Chest* **157**, 673–685 (2020). 31542452 10.1016/j.chest.2019.09.004

[36] Hansrivijit, P., Puthenpura, M. M., Ghahramani, N., Thongprayoon, C. & Cheungpasitporn, W. Bidirectional association between chronic kidney disease and sleep apnea: A systematic review and meta-analysis. *Int. Urol. Nephrol.* **53**, 1209–1222 (2021). 33155087 10.1007/s11255-020-02699-1

[37] Teng, Y., Wang, S., Wang, N. & Li, M. STOP-Bang questionnaire screening for obstructive sleep apnea among Chinese patients with type 2 diabetes mellitus. *Arch. Med. Sci. AMS* **14**, 971–978 (2018). 30154877 10.5114/aoms.2018.73984 PMC6111350

[38] Bernhardt, L. et al. Diagnostic accuracy of screening questionnaires for obstructive sleep apnoea in adults in different clinical cohorts: A systematic review and meta-analysis. *Sleep Breath.* **26**, 1053–1078 (2022). 34406554 10.1007/s11325-021-02450-9 PMC8370860

[39] Pecotic, R., Dodig, I. P., Valic, M., Ivkovic, N. & Dogas, Z. The evaluation of the Croatian version of the Epworth sleepiness scale and STOP questionnaire as screening tools for obstructive sleep apnea syndrome. *Sleep Breath.* **16**, 793–802 (2012). 21874368 10.1007/s11325-011-0578-x

[40] Chung, K. F. Use of the Epworth sleepiness scale in Chinese patients with obstructive sleep apnea and normal hospital employees. *J. Psychosom. Res.* **49**, 367–372 (2000). 11164062 10.1016/s0022-3999(00)00186-0

[41] Senaratna, C. V. et al. Detecting sleep apnoea syndrome in primary care with screening questionnaires and the Epworth sleepiness scale. *Med. J. Aust.* **211**, 65–70 (2019). 31049990 10.5694/mja2.50145

[42] Fernandez-Mendoza, J. et al. Natural history of excessive daytime sleepiness: Role of obesity, weight loss, depression, and sleep propensity. *Sleep* **38**, 351–360 (2015). 25581913 10.5665/sleep.4488 PMC4335535

[43] Mazzotti, D. R. et al. Symptom subtypes of obstructive sleep apnea predict incidence of cardiovascular outcomes. *Am. J. Respir. Crit. Care Med.* **200**, 493–506 (2019). 30764637 10.1164/rccm.201808-1509OC PMC6701040

[44] Shi, H. et al. Development and validation of a nomogram for predicting the risk of obstructive sleep apnea in patients with type 2 diabetes. *Ann. Transl. Med.* **8**, 1675–1675 (2020). 33490187 10.21037/atm-20-6890 PMC7812169

[45] McEvoy, R. D. et al. CPAP for prevention of cardiovascular events in obstructive sleep apnea. *N. Engl. J. Med.* **375**, 919–931 (2016). 27571048 10.1056/NEJMoa1606599

[46] Yang, D., Li, L., Dong, J., Yang, W. & Liu, Z. Effects of continuous positive airway pressure on cardiac events and metabolic components in patients with moderate to severe obstructive sleep apnea and coronary artery disease: A meta-analysis. *J. Clin. Sleep Med.* **19**, 2015–2025 (2023). 37497624 10.5664/jcsm.10740 PMC10692926

[47] Westlake, K., Dostalova, V., Plihalova, A., Pretl, M. & Polak, J. The clinical impact of systematic screening for obstructive sleep apnea in a type 2 diabetes population—Adherence to the screening-diagnostic process and the acceptance and adherence to the CPAP therapy compared to regular sleep clinic patients. *Front. Endocrinol.* **9**, 714 (2018). 10.3389/fendo.2018.00714 PMC6282364 30555416

[48] Foster, G. D. et al. Obstructive sleep apnea among obese patients with type 2 diabetes. *Diabetes Care* **32**, 1017–1019 (2009). 19279303 10.2337/dc08-1776 PMC2681024

[49] Resnick, H. E. et al. Diabetes and sleep disturbances: Findings from the Sleep Heart Health Study. *Diabetes Care* **26**, 702–709 (2003). 12610025 10.2337/diacare.26.3.702

[50] Amin, A. et al. Prevalence and associations of obstructive sleep apnea in South Asians and White Europeans with type 2 diabetes: A cross-sectional study. *J. Clin. Sleep Med.* **13**, 583–589 (2017). 28162147 10.5664/jcsm.6548 PMC5359335

[51] Leong, W. B. et al. The prevalence and severity of obstructive sleep apnea in severe obesity: The impact of ethnicity. *J. Clin. Sleep Med.* **09**, 853–858 (2013). 10.5664/jcsm.2978 PMC3746711 23997696

[52] Ejnell, H. et al.* National guidelines for the investigation of suspected sleep apnea in adults [Nationella riktlinjer för utredning av misstänkt sömnapné hos vuxna]*. (Swedish Sleep Apnea Registry (SESAR), 2018).

